# The telomere-to-telomere genome assembly of the *Triplophysa erythraea* (Nemacheilidae hypogean fishes)

**DOI:** 10.1038/s41597-025-06237-5

**Published:** 2025-12-10

**Authors:** Chongrui Wang, Xin Yang, Dong Liu, Wenwen Suo, Hong Li, Jinhui Pu, Xiangrong Liu, Qianqian Ku, Xiping Yuan, Yuejuan Zhou, Qianhong Gu, Dongsheng Ou

**Affiliations:** 1 Hunan Fisheries Research Institute and Aquatic Products Seed Stock Station, Changsha, 410153 People’s Republic of China; 2 College of Fisheries, Hunan Agricultural University, Changsha, 410128 People’s Republic of China; 3 Engineering Research Center of Polyploid Fish Reproduction and Breeding of the State Education, Ministry, College of Life Sciences, Hunan Normal University, Changsha, 410081 People’s Republic of China

**Keywords:** Conservation genomics, Genome assembly algorithms

## Abstract

*Triplophysa erythraea*, a distinctive blind cave fish endemic to Hunan’s karst caves, faces significant threats due to its restricted range and fragile habitat. The high-quality genome assembly offers essential data for developing conservation strategies, and could be helpful to reveal adaptive mechanisms along with functional drivers behind *T. erythraea*’s unique morphological traits. In this study, we successfully assembled the telomere-to-telomere (T2T) genome of *T. erythraea*. The genome size was 757.23 Mb, with a contig N50 size of 27.63 Mb and a scaffold N50 size of 29.01 Mb. The Hi-C assembly placed 97.5% of the sequences onto 25 pseudo-chromosomes. Remarkably, 19 chromosomes were assembled into contiguous, gap-free scaffolds. Furthermore, 378.05 Mb (49.93%) of repetitive sequences and 25,179 protein-coding genes were identified, and 99.09% of the protein-coding genes were annotated. Comparative genomic analysis confirmed the genome’s high completeness, continuity, and accuracy. The genomic quality was further substantiated by a QV of 51.03 and a 98.38% of BUSCO completeness rate, assessed against the *T. erythraea* T2T genome assembly. This study serves as a key genetic resource for Nemacheilidae hypogean fishes and will be highly valuable for delineating adaptive genetic mechanisms of cave-adapted *Triplophysa* stone loaches.

## Background & Summary

Major shifts in paleogeoclimatic have repeatedly driven biotic community reshaping, leading to rapid lineage divergence that shapes current biodiversity patterns^1–7^. The uplift of the Tibetan Plateau and the intensification of the East Asian monsoon drove the formation of eastward-flowing rivers like the Yangtze, fostering high cyprinid diversity and rapid speciation in East Asian lineages^8^. The phylogeographic dynamics of cyprinid fishes in turn provided crucial evidence for understanding the spatiotemporal evolution of their fluvial systems, including river connectivity, drainage changes, and habitat fragmentation^8,9^. Meanwhile, the rapid uplift of the Tibetan Plateau and the marked intensification of the East Asian monsoon system synergistically drove the formation of East Asia’s unique natural landscapes and hydrological regimes, exemplified by karst topography^10–12^. And the South China Karst has received broad recognition from the scientific community for its importance in biodiversity conservation^10,13^, as Karst regions’ abundant underground river networks have nurtured diverse cave fishes^14–16^. In recent decades, cave fishes have been continuously discovered since 1976, with the Nemacheilidae’s hypogean fishes representing nearly 90 described species^15,16^. Recent studies link cavefish diversification and speciation to paleoclimate shifts, especially the Tibetan Plateau uplift and East Asian monsoon intensification^15,17,18^, making them ideal models for exploring how these changes shaped karst biodiversity and subterranean ecosystem evolutionary mechanisms. However, current evidence remains inadequate to resolve the evolutionary mechanisms driving rapid radiation in cave-adapted *Triplophysa* or their adaptive strategies for surviving extreme subterranean environments. It is crucial to note that the fragility of karst landscapes (e.g., susceptibility to cave collapses and groundwater pollution in underground rivers) necessitates prioritizing ecological conservation in human activities^19–21^. Therefore, large-scale phylogeographic dynamic studies on cave-adapted fishes face significant challenges in reconstructing the spatiotemporal evolution of their subterranean river systems. However, cave-adapted fishes hold significant conservation value, making research into their survival mechanisms and adaptive evolutionary strategies in unique subterranean environments particularly crucial^16,22–24^. The Telomere-to-Telomere (T2T) genome enables the resolution of structural variations in complex genomic regions, the discovery of novel genes, and the functional annotation of “genomic dark matter” regions (e.g., centromeres and telomeres)^25–27^. These breakthroughs are critical for elucidating how species adapt to environmental changes.

**Fig. 1:**
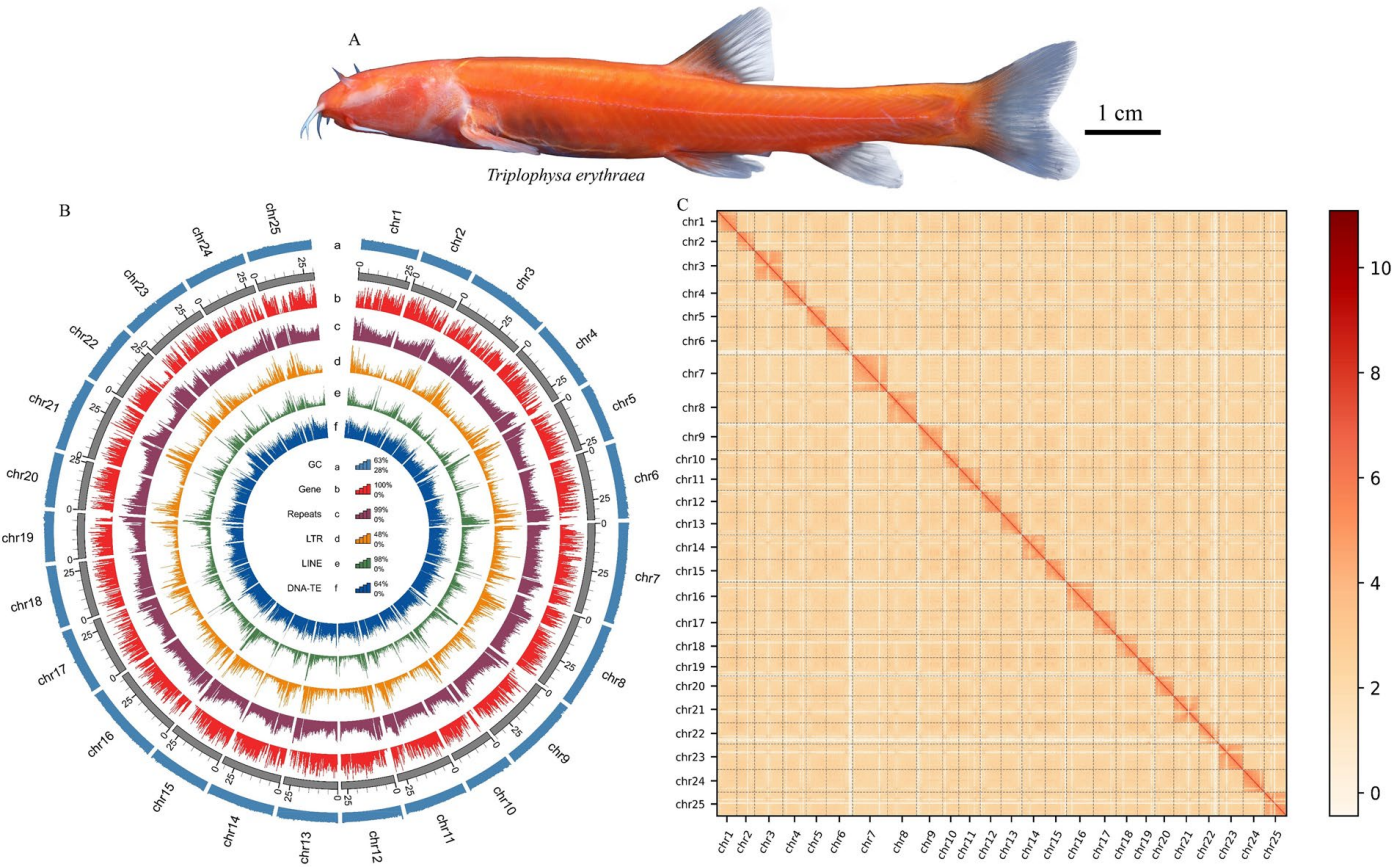
(**A**) *Triplophysa erythraea*, and the (**B**) circos plot illustrating the genome of the *T. erythraea*. The rings, from the outermost to the innermost layer, represent GC content (**a**), gene density (**b**), Repeats density (**c**), LTR density (**d**), LINE density (**e**), and DNA-TE density (**f**). The analysis was conducted using 300-kb genomic windows. (**C**) Chromosomal Hi-C heatmap of the *T. erythraea* genome assembly.

*Triplophysa erythraea* (Fig. 1A), a newly described cavefish species identified in 2019, exhibits extreme troglomorphic adaptations: complete absence of eyes, scaleless body, transparent integument, blood-red trunk pigmentation, and elongated barbels^28^. This remarkable species inhabits subterranean rocky pools at depths of 0.3–1.0 m, representing a significant taxonomic addition to the cavefish of South China. Cave-dwelling *Triplophysa* exhibit troglomorphic traits while retaining genetic similarities to epigean congeners within this genus^15,29^. This unique ecological specialization, which merges extreme subterranean adaptation with conserved genetic traits from surface-dwelling congeners, makes this clade an exemplary model for exploring the evolutionary mechanisms of cavefish adaptation.

In this study, we achieved the first chromosome-level, telomere-to-telomere (T2T) genome assembly for *T. erythraea* through integration of Pacific Biosciences (PacBio) HiFi sequencing, Oxford Nanopore Technologies (ONT) ultra-long sequencing, and Hi-C assisted assembly technology. This genomic resource bridges critical knowledge gaps by providing the first high-quality chromosome-level assembly for this species, while also advancing evolutionary insights into cave adaptation and informing genome-driven conservation strategies for imperiled subterranean fauna. Furthermore, this accomplishment provides vital genomic data for taxonomic and evolutionary studies within the Nemacheilidae family. It establishes a robust foundation for comparative genomics research on *Triplophysa* evolution, thereby enhancing our comprehension of how the uplift of the Tibetan Plateau, intensification of the East Asian monsoon, as well as the oscillations in Pleistocene glaciation influence the rapid radiation evolution of *Triplophysa* stone loaches.

## Methods

### Ethics statement

All experimental protocols utilized in this study have been approved by the Laboratory Animal Ethics Committee of the Centre for Applied Aquatic Genomics at the Chinese Academy of Fishery Sciences. The sample collection process complied with the guidelines of Chinese Academy of Fishery Sciences.

### Sample collection and processing

In present study, two *T. erythraea* individuals were sampled from the Underground River in Dalong Cave, Huayuan County, Xiangxi Tujia and Miao Autonomous Prefecture, Hunan Province, China. Tissue samples from *T. erythraea* were harvested and promptly preserved in liquid nitrogen until DNA or RNA extraction could be performed. Multiple tissues (muscle, brain, skin, gill, intestinal, pectoral fins, spleen, and heart) were collected, snap-frozen, and stored at −80 °C. Total RNA was extracted and used for transcriptome sequencing and genome annotation. Muscle tissue was specifically chosen for DNA and ultra-long ONT extraction and sequencing respectively. High molecular weight genomic DNA (gDNA) was extracted via SDS-based extraction, followed by QIAGEN^®^ genomic kit purification (Cat# 13343, QIAGEN) to ensure analytical-grade purity. Genomic DNA integrity and purity were validated by: (1) agarose gel electrophoresis (intact high-molecular-weight DNA without smearing), (2) NanoDrop™ UV-Vis spectrophotometry (concentration and purity via A260/A280/A230 ratios), and (3) Qubit™ fluorometry (high-sensitivity quantification).

High-quality RNA was extracted from all sampled tissues using TRIzol reagent (Invitrogen, MA, USA). RNA integrity (RIN > 8.0) and concentration (≥500 ng/μL) were validated via Agilent Bioanalyzer and Qubit™ assays. Poly-A selected RNA (10–15 μg/sample) was used for strand-specific library prep with NEBNext^®^ Ultra™ II Kit (NEB, USA), including UMIs to correct PCR duplicates. Indexed libraries were sequenced on Illumina NovaSeq. 6000 (PE150, 50 M reads/sample).

### Library preparation and sequencing

Firstly, the SMRTbell target library was meticulously prepared in strict compliance with the established protocol (Pacific Biosciences, CA, USA). Subsequently, genome sequencing was performed in OneMore-tech Co.,Ltd. (Wuhan, China) using three complementary approaches: (1) PacBio HiFi reads (10-50 kb insert size) were generated from SMRTbell libraries (v2.0) on the Sequel II system, yielding 124.92 Gb data with N50 = 20.21 kb; (2) Oxford Nanopore ultra-long reads (N50 = 100 kb) were obtained via SDS-based lysis protocol, generating 14.98 Gb sequences; (3) Hi-C libraries were constructed following Belton *et al*.‘s protocol^30^, producing 62.16 Gb clean data for phased assembly (Table 1).

**Table 1:** Statistics of sequencing reads data.

Libraries	Clean reads number	Clean data (Gb)	Average length (bp)	GC content (%)
PacBio SMRT	6,672,784	124.92	20,101	39.67
ONT ultra-long	104,889	14.98	153,329	40
Hi-C	447,891,714	62.16	150	41.34

### Genome assembly and gap filling

The initial hybrid genome assembly was performed using HiFiasm (v0.16.0) by integrating HiFi reads (PacBio), ONT ultra-long reads, and Hi-C contact maps^31^, achieving a draft genome of 760.43 Mb with a contig N50 of 27.53 Mb (Table 2). Chromosome-level assembly was achieved through Hi-C-based scaffolding, with quality-controlled Hi-C reads aligned to contig-level genomes using Bowtie2 (v2.3.4.3) under paired-end mode^32^, yielding 97.20 million uniquely mapped reads (43.41% valid inter-chromosomal pairs, Tables 3, 4). The 3D-DNA pipeline v180922 was employed for chromatin interaction frequency analysis and scaffolding error correction^33^, followed by iterative refinement using JuiceBox v1.11.08^34^. This integrative approach produced 25 pseudo-chromosomes spanning 97.5% of the genome assembly (contig N50 = 27.32 Mb) (Fig. 1C).

**Table 2:** Statistics for the *T. erythraea* preliminary genome assembly.

Mode	Total length (Mb)	Total number	N50 (Mb)	N90 (Mb)	GC content (%)
Hifiasm (CCS + ONT + Hi-C)	760	122	28	14	40

**Table 3:** Statistics of alignment results of clean paired-end reads.

	Read 1		Read 2	
	Number	Percentage (%)	Number	Percentage (%)
Unique alignments	47,976,226	21.42	43,999,426	19.64
Multiple alignments	53,298,980	23.80	56,478,095	25.224
Too short to align	15,349,778	6.85	15,761,350	7.04
Filed to align	10,116,335	4.52	10,502,448	4.69
Paired	97,204,538	43.41	97,204,538	43.41

**Table 4:** Statistics of valid paired-end reads.

	Di-Tag Count	Percent in Paired (%)	Percent in Total Reads(%)
Same Circularised	192,917	0.2	0.09
Same Fragment Dangling Ends	895,743	0.92	0.4
Same Fragment Internal	5,001,879	5.15	2.23
Re-ligation	3,666,445	3.77	1.64
Contiguous Sequence	0	0	0
Wrong Size	0	0	0
Invalid Pairs	9,756,984	10.04	4.36
Valid Pairs	87,447,554	89.96	39.05
Valid Pairs (de-duplication)	87,096,048	89.6	38.89

**Fig. 2:**
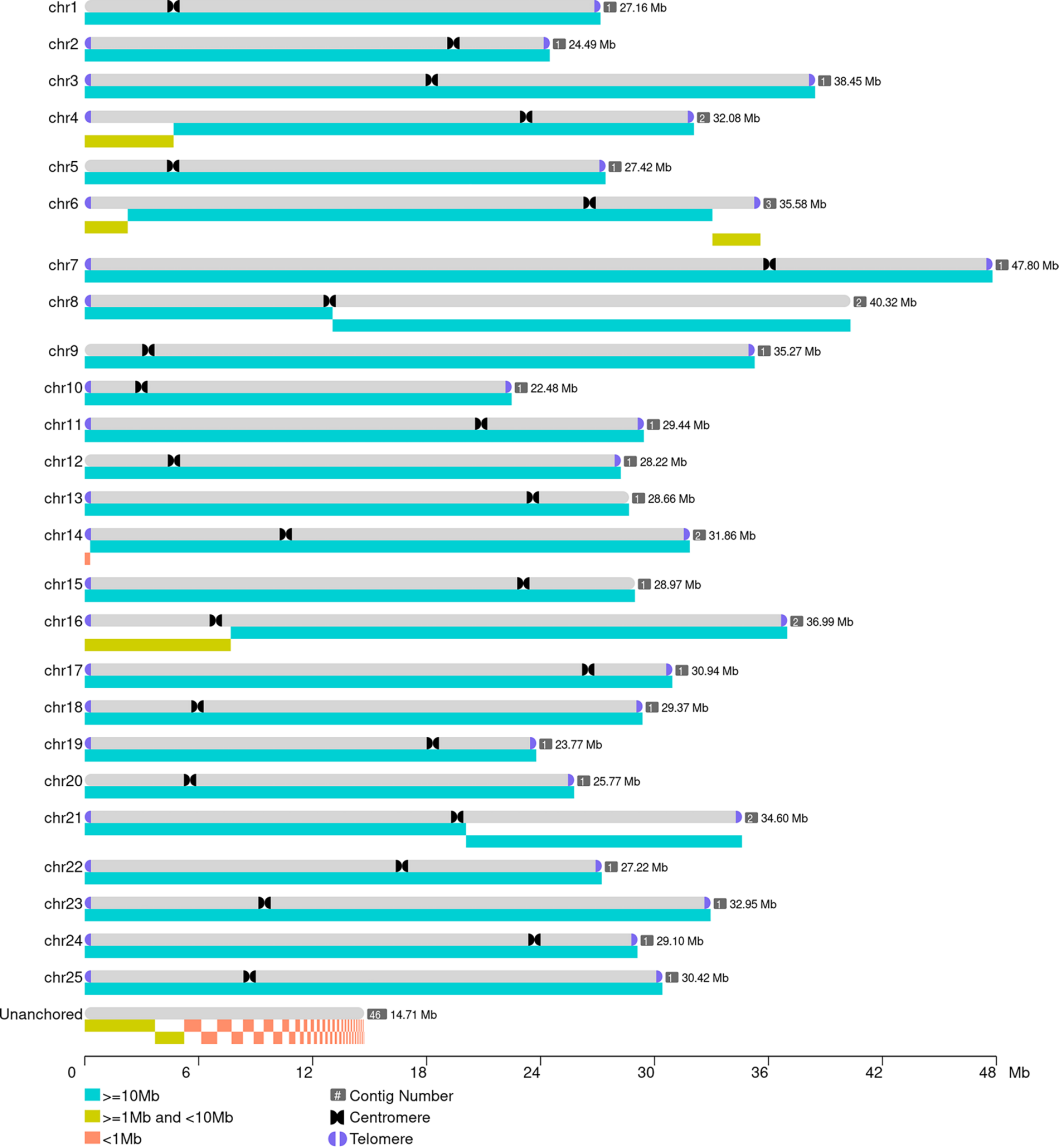
The contigs in the chromosomes of the *T. erythraea* genome. The blue sections at each end of chromosomes and black sections inside each chromosomes represent the identified telomeres and centromeres respectively.

The T2T genome assembly was accomplished through a multi-step workflow: (1) ONT ultra-long reads were mapped to pseudo-chromosomes using minimap2^35^ with–secondary = no flag to exclude multi-mapping artifacts; (2) TGS-GapCloser v1.1.1^36^ executed gap filling by leveraging long-read continuity; (3) iterative refinement was performed via three Pilon v1.24^37^ correction cycles. This pipeline produced a 757.23 Mb telomere-to-telomere genome (contig N50 = 27.63 Mb) containing 19 fully resolved chromosomes (Fig. 2, Table 5), achieving 98.38% completeness as validated by Merqury (QV = 51.03).

**Table 5:** Statistics for assembled chromosome, telomeres and centromeres.

Chromosomes	Length (bp)	Number of contigs	Number of gaps	Number of telomeres (Front)	Number of telomeres (End)	Centromeric region start (bp)	Centromeric region end (bp)
chr1	27,155,284	1	0	0	69	4672982	4984765
chr2	24,485,445	1	0	1584	889	19412292	19946959
chr3	38,454,566	1	0	1189	1476	18268512	19375242
chr4	32,075,147	2	1	1609	789	23239371	24038192
chr5	27,419,522	1	0	0	667	4650793	5085457
chr6	35,575,382	3	2	823	111	26581229	27559306
chr7	47,795,519	1	0	802	1088	36055128	37729918
chr8	40,318,098	2	1	1157	0	12895149	13475284
chr9	35,274,438	1	0	0	1028	3346195	4545956
chr10	22,476,330	1	0	994	1058	2984986	4488781
chr11	29,440,139	1	0	385	959	20872326	21673275
chr12	28,224,040	1	0	0	1337	4696851	5342909
chr13	28,657,973	1	0	28	0	23593521	24195025
chr14	31,861,538	2	1	1098	2020	10585396	11335185
chr15	28,966,947	1	0	844	0	23093180	23408899
chr16	36,986,285	2	1	1234	1721	6900373	8448353
chr17	30,937,995	1	0	1058	1306	26507048	26895953
chr18	29,370,829	1	0	1127	1058	5934112	6295968
chr19	23,772,279	1	0	1254	766	18326732	19322058
chr20	25,767,922	1	0	0	1093	5543613	6064634
chr21	34,602,359	2	1	1068	1944	19610585	21096274
chr22	27,219,775	1	0	1414	1310	16700265	17844467
chr23	32,950,521	1	0	126	2242	9467186	11550101
chr24	29,102,906	1	0	1228	1130	23676709	24445934
chr25	30,416,603	1	0	1568	1172	8681519	10222497

### Telomere and centromeric regions analysis

Telomere and centromere characterization was conducted using quarTeT v1.1.4^38^, a specialized toolkit for T2T genome analysis. Telomere detection employed motif scanning to identify TTAGGG/CCCTAA repeats with a minimum of four contiguous units, leveraging the TeloExplorer module’s optimized threshold algorithms. Centromere prediction integrated genome annotations with automated tandem repeat detection through the CentroMiner module, which clusters satellite DNAs (≥5 repeats) and prioritizes regions with >72% repeat density. This pipeline generated 42 telomeric regions (17 pairs) and 19 centromere candidates across all chromosomes (Fig. 2, Table 5).

### Repetitive sequences analysis

The repetitive landscape was characterized using *de novo* (RepeatModeler v1.0.11 + LTR-FINDER_parallel v1.0.7) and homology-based (RepeatMasker v4.09 + TRF v4.09) approaches^39–42^, revealing 378.05 Mb repetitive sequences (49.93% genome coverage) dominated by 23.83% DNA transposons, 6.93% LINEs, and 8.99% LTR retrotransposons (Table 6, Fig. 1B). Full annotations, including element distribution and evolutionary dynamics, are detailed in Table 7. Repetitive sequences comprise nearly half of the genome, a notable feature given their established roles in shaping genome stability, modulating gene expression, and generating phenotypic diversity. These functions are critical for understanding the molecular basis of adaptation in *T. erythraea* to extreme environments.

**Table 6:** Transposable elements statistics for the *T. erythraea* genome.

Type	Repbase TEs		TE protiens		De novo		Combined TEs	
	Length (bp)	Percentage of genome (%)	Length (bp)	Percentage of genome (%)	Length (bp)	Percentage of genome (%)	Length (bp)	Percentage of genome (%)
DNA	110,018,513	13.86	8,882,742	1.12	125,893,982	15.86	18,921,4617	23.83
LINE	39,576,077	4.98	31,284,014	3.94	34,893,050	4.39	54,998,357	6.93
SINE	4,839,836	0.61	0	0	2,315,703	0.29	6,717,720	0.85
LTR	42,831,560	5.39	27,763,515	3.5	50,745,746	6.39	71,394,596	8.99
Satellite	3,002,556	0.38	0	0	1,464,292	0.18	4,346,240	0.55
Simple_repeat	0	0	0	0	58,689	0.01	58,689	0.01
Other	1,041	0	0	0	0	0	1,041	0
Unknown	2,685,782	0.34	2,454	0	50,138,160	6.31	52,498,262	6.61
Total	194,596,069	24.51	67,906,621	8.55	262,308,848	33.04	362,559,718	45.66

**Table 7:** Repetitive sequences statistics for the *T. erythraea* genome.

Type	Repeat size (bp)	Percentage of genome (%)
Trf	58,062,506	7.31
Repeatmasker	194,596,069	24.51
Proteinmask	67,906,621	8.55
De novo	262,308,848	33.04
Total	396,419,145	49.93

### Prediction and functional annotation of protein-coding genes

Genome assembly of *T. erythraea* underwent comprehensive ab initio gene prediction using a multi-tool pipeline. *De novo* predictors included AUGUSTUS v3.3.2^43^ for specific splicing patterns, Genscan v1.0 for gene architectures and GlimmerHMM v3.0.4^44,45^ for prokaryotic-derived eukaryotic gene models. Evidence-based refinement employed GeneWise v2.4.1^46^ to align homologous proteins with E-value ≤ 1e^-10^, resolving splice junctions with ≤5% false discovery rate.

Transcriptomic validation integrated RNA-seq data (Illumina NovaSeq. 6000) using HISAT2 v2.2.1^47^ with–dta flag for splice-aware alignment, followed by StringTie v2.2.0^48^ for transcript quantification and PASA v2.3.2^49^ for consensus isoform assembly. Hybrid annotation merged these predictions via MAKER2 v2.31.10^50^ and HiFAP, generating 25,179 protein-coding genes (Table 8) with 97.69% BUSCO completeness.

**Table 8:** Statistics on transposable elements in the *T. erythraea* genome.

	Gene set	Protein coding gene number	Average gene length (bp)	Average CDS length (bp)	Average exon per gene	Average exon length (bp)	Average intron length (bp)
De novo	Genscan	28,740	18,167	1,611	8.52	189.16	2,203
	AUGUSTUS	24,438	11,953	1,429	8.27	172.83	1,448
Homolog	*Triplophysa yaopeizhii*	42,491	10,216	1,376	6.9	199.5	1,499
	*Triplophysa tibetana*	28,682	12,763	1,533	8.11	189.02	1,580
	*Triplophysa dalaica*	53,849	18,833	1,886	10.73	175.85	1,743
	*Triplophysa rosa*	52,283	19,422	1,973	10.96	180.04	1,752
	*Ctenopharyngodon idella*	68,558	23,890	2,117	11.42	185.39	2,090
Liftoff	*Triplophysa rosa*	23,335	14,708	1,744	9.97	174.87	1,444
	*Triplophysa dalaica*	22,752	13,947	1,707	9.82	173.86	1,388
Trans ORF	RNAseq	14,802	18,387	1,856	11.76	304.64	1,376
	BUSCO	3,663	11,469	1,633	10.23	159.61	1,065
	MAKER	25,593	15,577	1,521	9.63	280.24	1,493
	HiFAP	25,179	15,309	1,669	9.72	252.31	1,475

**Fig. 3:**
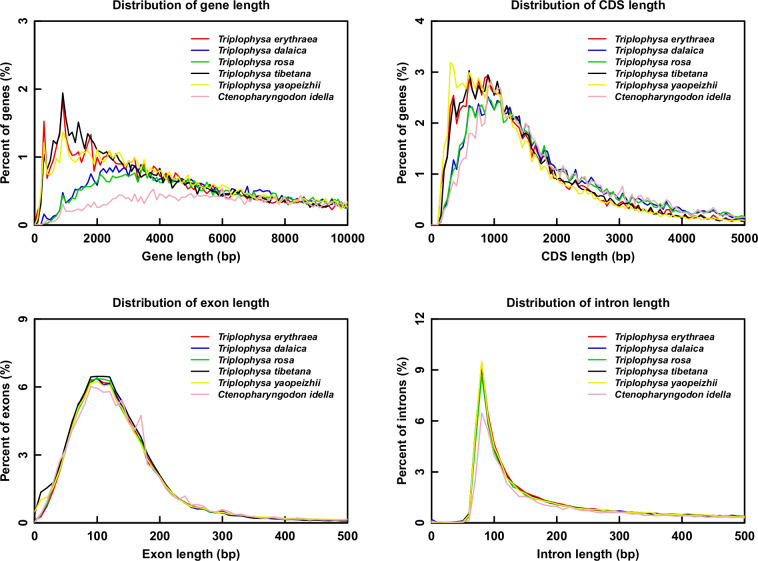
Distribution of genes in different related species.

TBLASTN-based comparative genomics (E-value ≤ 1e^-5^) identified 3,663 conserved coding regions across the related species^51^, including *Triplophysa yaopeizhii*, *T. tibetana*, *T. dalaica*, *T. rosa*, and *Ctenopharyngodon idella*. The gene structures were compared and juxtaposed with those of homologous species, as depicted in Fig. 3. As shown in Fig. 3, the four dimensions exhibit high intra-genus consistency in gene structure across the five *Triplophysa* species. Notably, distinct differences from the outgroup (*C. idella*) are evident in two key aspects, including shorter overall gene length and shorter introns. Shorter genes and introns contribute to enhanced transcriptional efficiency, while the relatively shorter coding sequences (CDSs) and exons help maintain stable gene function^52^. Collectively, these structural features could be helpful to the survival and reproduction of *Triplophysa* in low-temperature and hypoxic environments.

Comprehensive functional annotation of protein-coding genes was executed through iterative database curation using InterProScan v5.61–93.0^53^ for conserved domain/motif detection (99.09% annotated genes, 24,951 entries), followed by InterPro, GO, KEGG, and SwissProt enrichment analysis^54–58^. Multi-source validation integrated TrEMBL (98.41% coverage)^58^, Pfam (85.95 domain overlap), and KOG (75.55% orthology groups), with TF and NR databases resolving unannotated gene families (Table 9).

**Table 9:** Putative protein-coding gene functional annotations of the *T. erythraea* genome.

		Database	Annotated number of putative genes	Percent (%)
Total			25,179	100
	Annotated		24,951	99.09
		NR	24,906	98.92
		SwissProt	20,670	82.09
		TrEMBL	24,778	98.41
		KOG	19,022	75.55
		TF	6,108	24.26
		InterPro	23,021	91.43
		GO	17,207	68.34
		KEGG_ALL	24,366	96.77
		KEGG_KO	16,215	64.4
		Pfam	21,642	85.95
	Unannotated		228	0.91

### Annotation of non-coding RNAs

Non-coding RNA annotation was performed using specialized bioinformatics pipelines. The tRNA was identified using tRNAscan-SE v1.3.1^59^ with E-value cutoff ≤ 1e^-5^. The rRNA was predicted by BLASTN alignment. Additionally, the miRNA and snRNA were identified via INFERNAL v1.1.4 trained on Rfam v14.8^60^, and the results were summarized in Table 10. Different non-coding RNAs vary widely in genomic abundance. For example, rRNA (especially 18S) account for a higher genomic proportion (0.025811%), while scaRNAs represent a far smaller fraction (0.000281%). This marked disparity likely reflects their distinct functional roles and biological significance in *T. erythraea*.

**Table 10:** Statistics of the noncoding RNA in the *T. erythraea* genome.

Type		Copy	Average length(bp)	Total length(bp)	% of genome
miRNA		1,723	89	153,319	0.019309
tRNA		13,388	74	987,709	0.124394
rRNA	rRNA	4,676	162	755,885	0.095197
	18S	125	1,640	204,946	0.025811
	28S	0	0	0	0
	5.8S	106	152	16159	0.002035
	5S	4445	120	534780	0.067351
snRNA	snRNA	2228	146	325247	0.040962
	CD-box	336	171	57476	0.007239
	HACA-box	73	149	10909	0.001374
	splicing	1809	141	254633	0.032069
	scaRNA	10	223	2229	0.000281

## Data Records

The raw sequencing reads generated from three platform-specific sequencing runs, along with the final genome assembly, have been deposited in the NCBI Sequence Read Archive (SRA, accession number: SRR34067827 - SRR34067831) under BioProject accession number PRJNA1279685^61–65^. The genome annotation files are available in figshare: 10.6084/m9.figshare.29367860^66^.

## Technical Validation

Genome assembly validation was performed through multi-platform read alignment. The workflow achieved 99.69% alignment rate for short reads using BWA-MEM (v0.7.17, r = 1188)^67^, and 99.94% and 99.91% mapping rates for HiFi and ONT reads via Minimap2 v2.24^35^), respectively (Tables 11, 12). This dual-validation strategy demonstrated exceptional genomic congruence, with BUSCO v5.4.3^68^ analysis (actinopterygii_odb10) revealing 98.38% completeness across 3,581 single-copy orthologs (Table 13).

**Table 11:** The alignment of the short reads to the *T. erythraea* genome assembly.

Mapping Rate (%)	Paired mapping rate (%)	Average sequencing depth	Coverage (%)	Coverage at least 4× (%)	Coverage at least 10× (%)	Coverage at least 20× (%)
99.69	98.99	100.81	99.86	99.68	99.43	98.97

**Table 12:** The alignment of the long reads to the *T. erythraea* genome assembly.

Mapping Rate (%)	Average sequencing depth	Coverage (%)	Coverage at least 4× (%)	Coverage at least 10× (%)	Coverage at least 20× (%)
99.94	168.31	99.79	99.67	98.51	99.24

**Table 13:** Statistics of BUSCO analysis of the *T. erythraea* genome.

Type	Assembly		Annotation	
	Proteins	Percentage (%)	Proteins	Percentage (%)
Complete BUSCOs	3,581	98.38	3,556	97.69
Complete Single-Copy BUSCOs	3,514	96.54	3,465	95.19
Complete Duplicated BUSCOs	67	1.84	91	2.5
Fragmented BUSCOs	22	0.6	10	0.27
Missing BUSCOs	37	1.02	74	2.03
Total BUSCO groups searched	3,640	100	3,640	100

## Data Availability

The chromosome-level genome assembly of *Triplophysa erythraea* has been deposited in the National Center for Biotechnology Information (NCBI) GenBank database under the accession number JBQWDH000000000^69^.

## Code availability

Data processing pipelines and commands were executed strictly following bioinformatics software protocols, with no custom code developed for this study.
